# Protein Biomarkers in Blood Reflect the Interrelationships Between Stroke Outcome, Inflammation, Coagulation, Adhesion, Senescence and Cancer

**DOI:** 10.1007/s10571-022-01260-1

**Published:** 2022-08-11

**Authors:** Georg Fuellen, Uwe Walter, Larissa Henze, Jan Böhmert, Daniel Palmer, Soyoung Lee, Clemens A. Schmitt, Henrik Rudolf, Axel Kowald

**Affiliations:** 1 Institute for Biostatistics and Informatics in Medicine and Ageing Research, Rostock University Medical Center and Centre for Transdisciplinary Neurosciences Rostock and Research Focus Oncology, Rostock and Ageing of Individuals and Society, Interdisciplinary Faculty, Rostock University, Rostock, Germany; 2 Department of Neurology, Rostock University Medical Center and Centre for Transdisciplinary Neurosciences Rostock, Rostock, Germany; 3 Department of Medicine, Clinic III, Hematology, Oncology, Palliative Medicine, Rostock University Medical Center and Research Focus Oncology, Rostock, Germany; 4 Medical Department of Hematology, Oncology and Tumor Immunology, and Molekulares Krebsforschungszentrum – MKFZ, Charité - Universitätsmedizin, Campus Virchow Klinikum, Augustenburger Platz 1, 13353 Berlin, Germany; 5 Max-Delbrück-Center for Molecular Medicine in the Helmholtz Association, Robert-Rössle-Straße 10, 13125 Berlin, Germany; 6 Johannes Kepler University, Altenbergerstraße 69, 4040 Linz, Austria; 7 Department of Hematology and Oncology, Kepler University Hospital, Krankenhausstraße 9, 4021 Linz, Austria; 8 Institute of Tumor Biology, Kepler University Hospital, Krankenhausstraße 9, 4021 Linz, Austria

**Keywords:** Cellular senescence, Aging, Cancer, Coagulation, Inflammation

## Abstract

The most important predictors for outcomes after ischemic stroke, that is, for health deterioration and death, are chronological age and stroke severity; gender, genetics and lifestyle/environmental factors also play a role. Of all these, only the latter can be influenced after the event. Recurrent stroke may be prevented by antiaggregant/anticoagulant therapy, angioplasty of high-grade stenoses, and treatment of cardiovascular risk factors. Blood cell composition and protein biomarkers such as C-reactive protein or interleukins in serum are frequently considered as biomarkers of outcome. Here we aim to provide an up-to-date protein biomarker signature that allows a maximum of mechanistic understanding, to predict health deterioration following stroke. We thus surveyed protein biomarkers that were reported to be predictive for outcome after ischemic stroke, specifically considering biomarkers that predict long-term outcome (≥ 3 months) and that are measured over the first days following the event. We classified the protein biomarkers as immune‑inflammatory, coagulation-related, and adhesion-related biomarkers. Some of these biomarkers are closely related to cellular senescence and, in particular, to the inflammatory processes that can be triggered by senescent cells. Moreover, the processes that underlie inflammation, hypercoagulation and cellular senescence connect stroke to cancer, and biomarkers of cancer-associated thromboembolism, as well as of sarcopenia, overlap strongly with the biomarkers discussed here. Finally, we demonstrate that most of the outcome-predicting protein biomarkers form a close-meshed functional interaction network, suggesting that the outcome after stroke is partially determined by an interplay of molecular processes relating to inflammation, coagulation, cell adhesion and cellular senescence.

## Introduction

Ischemic stroke (stroke for short) biomarkers are an active and important area of research. The reduction in quality-of-life and in life expectancy after stroke, combined with its limited treatment options, are all responsible for the high socio-economic burden of the disease (Uivarosan et al. 2020). Thus, primary and secondary prevention is important, and secondary prevention should be based on valid predictors of disease progression (including recurrent stroke). It is commonplace to delineate predictors into risk factors such as chronological age and smoking, and (molecular) biomarkers such as lipid profiles. Here, we do not consider the risk factors or biomarkers for a primary stroke event; we refer to, e.g., (Donkor 2018), which also discusses primary prevention. Instead, in this narrative review, we consider outcomes after a stroke event, and present the associated risk factors and biomarkers. More specifically, our review focuses on biomarkers obtained during the acute stage of stroke (first days after stroke onset) and their association with chronic (≥ 3 months) outcome after stroke. Biomarkers related mainly to acute (≤ 7 days) and subacute (< 3 months) stroke outcome have been addressed in recent meta-analyses (Martin and Price 2018; Kim et al. 2022), these were not in the scope of our study. In terms of secondary prevention, we note several therapies including antiaggregant/anticoagulant therapy, angioplasty of high-grade stenoses of cerebral arteries, and medical treatment of cardiovascular risk. Timely administration of these therapies is particularly valuable if risk factors such as arteriosclerotic stenosis, atrial fibrillation, or dyslipidemia are present (Hankey 2014).

To tailor secondary prevention to the patient, biomarkers are essential. Optimally, these predict future disease, dysfunction or death, hint at underlying causal mechanisms, and predict the results of interventions. Biological age is a general predictor of (comorbid) disease and dysfunction (Fuellen et al. 2020). Estimated by an epigenetic (DNA methylation) assay, it contributes to outcome prediction after stroke (Soriano-Tarraga et al. 2021), and patients with a high biological age may be given more intensive care. Other predictors are based on gene expression (Sykes et al. 2021) and protein abundance (Montellano et al. 2021). Proteins are expected to be involved directly in the molecular processes that influence the outcome, so they are informative about both molecular mechanisms and intervention success rates, and therefore we focus on those in this review. The mechanistic role of proteins allows two important steps towards a better understanding of their function as biomarkers, and the progression of the disease: First, we can assign the proteins to specific molecular/cellular processes deemed important for the health deterioration observed after stroke. Here, we follow up upon the classification by Lehmann et al. (2021), considering immune‑inflammatory, coagulation-related, and adhesion-related proteins. Second, we can generate a protein interaction network from the proteins that describes their molecular interrelationships. Here, we employ the STRING (Jensen et al. 2009) database to describe how the proteins, and therefore the molecular/cellular processes they participate in, are intertwined.

## Data Collection and Tabulation: Blood Biomarkers of Stroke Relate to Immunity, Inflammation, Coagulation, and Adhesion

For this narrative review, we surveyed the literature on protein biomarkers for human ischemic stroke outcome prediction in adults, published from September 1, 2018 until July 30, 2021. We included recent reviews, which specifically cover the time before 2018, as well as the primary literature, to cover more recent developments. All papers had to include protein biomarkers; we also list biomarkers of other types if these are mentioned alongside the proteins. All biomarkers had to predict long-term (chronic, ≥ 3 months) outcome; their assignment to the categories of immune‑inflammation-related, coagulation-related, and adhesion-related was then based on the papers that report the markers, following the scheme by Lehmann et al. (Lehmann et al. 2021). Finally, we used the STRING database (Jensen et al. 2009) to exemplify a functional interaction network in which most of the protein biomarkers are involved. More specifically, we took all proteins from the table, submitted them to STRING, and captured the resulting network, as well as the gene ontology biological process annotation provided by STRING.

We first considered systematic reviews, from which we transcribed only the high-quality biomarkers into Table 1, that is, the biomarkers most consistently associated with outcome and (if available) with an added value compared to clinical routine (that is, compared to standard clinical and demographic markers that are routinely measured). We found no meta-analyses; this observation may be explained by the heterogeneity of the existing studies, according to (Montellano et al. 2021). In fact, that review is the most exhaustive recent systematic review, based on 291 studies, screened until August 2018, including biomarkers measured up to 7 days post-stroke, and predicting outcomes thereafter. As the abstract states, “natriuretic peptides, copeptin, procalcitonin, mannose-binding lectin, adipocyte fatty acid–binding protein, and cortisol were the biomarkers most consistently associated with poor outcome in higher-quality studies showing an incremental value over established prognostic factors”, where established factors refer to clinical routine. These high-quality biomarkers we report in Table 1 (top), together with a few “atherogenesis” biomarkers highlighted by the same authors and reported by higher-quality studies to be associated with poor outcome (but with no explicit reference to any incremental/added value). The authors summarize the roles of the biomarkers as “inflammation, atherogenesis, and stress response”, which overlaps with our classification of immune‑inflammation-related, coagulation-related, and adhesion-related biomarkers. Notably, they also report that CRP, TNFα and two cellular measurements [neutrophil–lymphocyte ratio (NLR) and white-blood cell count (WBC)] only feature inconsistent associations, while IL-6 is supported by two higher-quality studies of sufficient size, so this biomarker can still meet the “*most consistently associated in higher-quality studies showing an incremental value”* criterion.

**Table 1:**
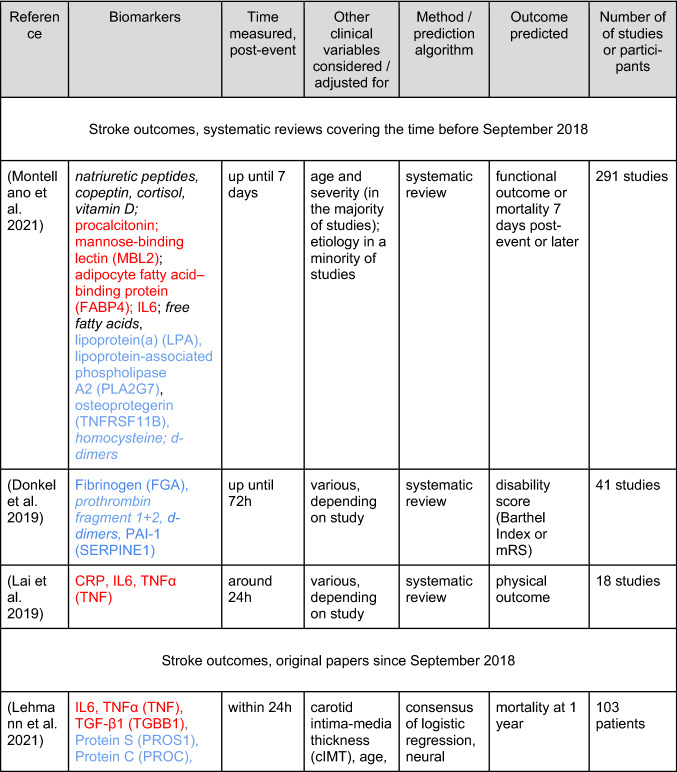

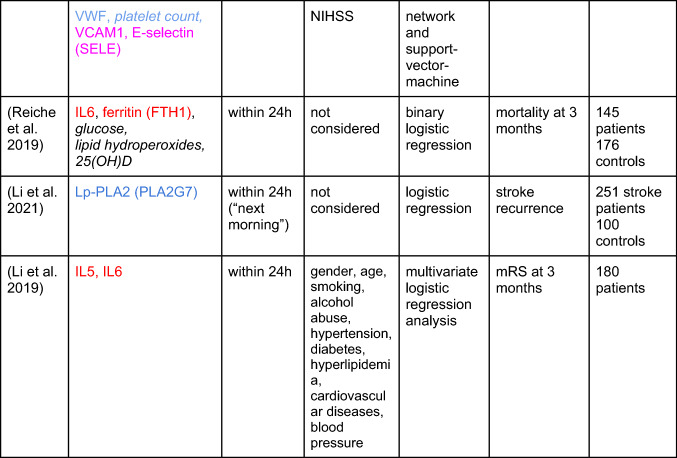
Blood-based biomarkers that predict stroke outcomes

Stringent criteria were applied to biomarkers reported in systematic reviews. HUGO gene names are given in parentheses where applicable. The color code of the biomarkers is red (immune-inflammatory), blue (coagulation-related) and magenta (adhesion-related). Non-protein biomarkers are given in italics

*mRS* modified Rankin scale

Another systematic review (Donkel et al. 2019), is based on 41 studies, screened until June 2018, with a focus on biomarkers of the hemostatic system (measured up until 72 h post-event) as predictors, and disability (not considering death) as the outcome, and we list their results in Table 1 as well. More specifically, we went through their main table and transcribed biomarkers with evidence from more than one study, and where 50% or more of these studies had to demonstrate an association with outcome, but not necessarily as an association with an added value (i.e., the association did not have to be demonstrated in, e.g., a multivariable regression analysis). Study quality was reported in the supplement, but all studies that met the inclusion criteria were included, irrespective of the quality score. The authors only consider biomarkers related to coagulation in the first place, without any further classification. A further publication (Lai et al. 2019) also provides a systematic review, based on 18 studies, screened until September 2018, with a focus on biomarkers measured around 24 h post-event, as predictors of physical function or physical function recovery (not considering death) as the outcome; their biomarkers were included in Table 1 if they were reported to be “[c]onsistently […] found to be robust predictors of long-term functional outcome in ischemic stroke” (Lai et al. 2019). Study quality is assessed by (Lai et al. 2019), but not used further in their analysis. The biomarkers are classified as related to immune response, lipids/metabolism (here they found no protein markers), neuronal function and blood vessel/circulation, again closely overlapping with our classification.

Next, using google scholar searches, we screened the primary literature from September 1, 2018 onwards, from which we transcribed biomarkers even if their added value was not established, into Table 1 (bottom). As noted by (Montellano et al. 2021), few original studies consider the added value of the biomarkers they investigate, by reporting prediction models that consider clinical routine data as well (often, not even age or stroke severity are considered), and it appears that the situation has not changed much since the August 2018 cutoff of (Montellano et al. 2021). Also, we did not formally assess study quality ourselves, and the overall number of participants is merely noted (though it is larger than 100 in all cases). Nevertheless, more recent studies usually feature more participants and, often, higher methodological quality, justifying the less strict criteria we imposed upon the recent primary literature.

In particular, from a 2019 study (Reiche et al. 2019), using the modified Rankin scale (mRS) as outcome, we only considered the predictor (not the association) analyses, based on logistic regression. We ignored the two composite scores reflecting inflammatory indices. The biomarkers were characterized as “immune-inflammatory, oxidative stress and biochemical”. In a 2021 study by an overlapping set of authors, (Lehmann et al. 2021), using 1-year mortality as outcome, the authors calculate predictors by logistic regression, neural network and support-vector-machine, and we took the consensus biomarkers as reported in the discussion section of the paper, considering these to be the most robust predictors. As already noted, these biomarkers were classified as belonging to “immune‑inflammatory, coagulation and adhesion” (carotid intima-media thickness was not part of the consensus), and we adopted this scheme as the blueprint for our review, since these also fit well with the biological processes ascribed to the biomarkers found in the three systematic reviews. We found a few more recent studies concerned with predicting outcome after stroke. One study (Li et al. 2021) confirmed Lp-PLA2, which was already noted by (Montellano et al. 2021). Finally, (Li et al. 2019) investigated cytokine profiles, and found IL5 and IL6 as independent predictors of outcome.

## Interaction Network and Gene Ontology Analysis of Blood Biomarkers of Stroke Corroborate Their Known Roles

**Fig. 1:**
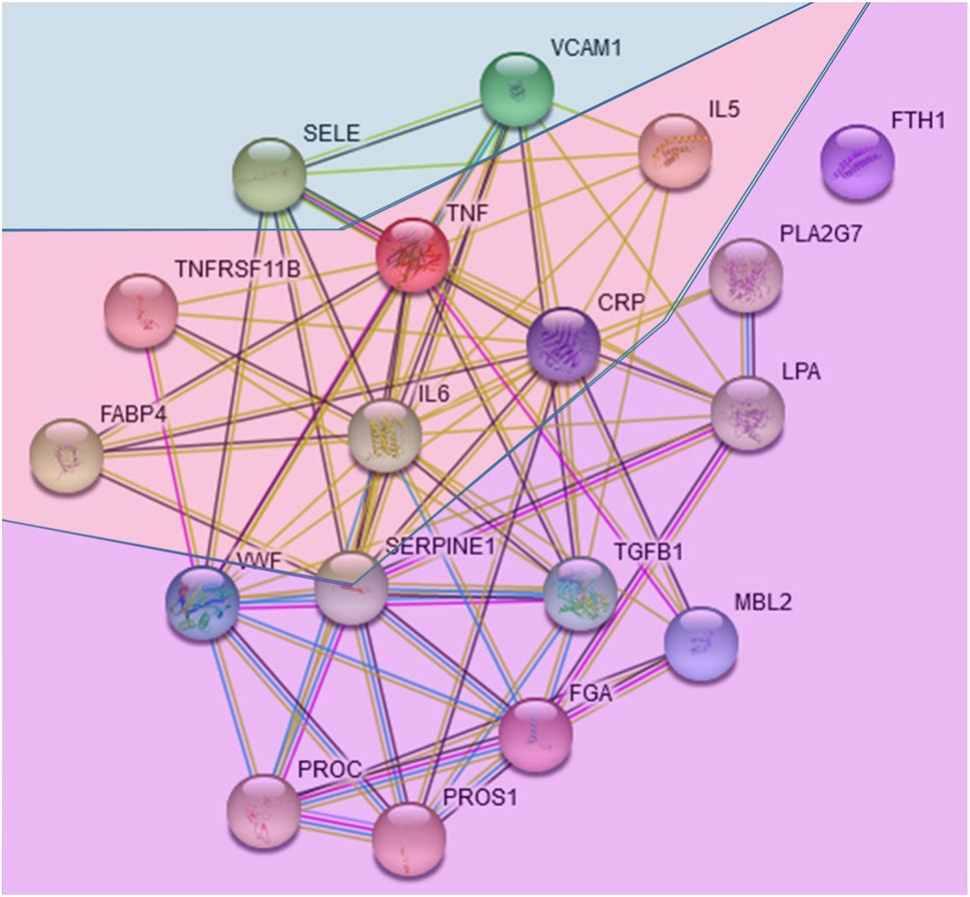
STRING network of the proteins in Table 1. The three adhesion-related biomarkers are positioned at the top; the coagulation-related biomarkers are positioned at the bottom. The immune-inflammation biomarkers are found in the middle of the network (pale red), using the default parameters of the STRING web interface; the layout considers node connectivity. As explained in detail in the online legend associated with the network, there is no particular meaning of the node color. The edge color refers to the source database of the interaction, i.e., curated databases (cyan), experimentally determined (magenta), predicted by gene neighborhood (green), by gene fusions (red) or by gene co-occurrence (blue), or taken from text mining (light green), co-expression (black) or protein homology (light blue) data. The permanent link to the network, including an online legend, is https://version-11-0b.string-db.org/cgi/network?taskId=b4MNWvbtbPcL

As described, the protein biomarkers for predicting outcome after ischemic stroke that we tabulated in this review (Table 1) can be assigned to the three classes of immune‑inflammatory, coagulation-related, and adhesion-related proteins, following the scheme of (Lehmann et al. 2021). Using the genes of Table 1 as input, a STRING functional interaction network was assembled, describing how these proteins are interacting, considering the default sources of evidence employed by STRING (Fig. 1). The network reflects the three classes, placing immune‑inflammatory proteins into the center of the network. PAI-1/SERPINE1 appears as a hub, reflecting its involvement in inflammation as well as coagulation (Hisada and Mackman 2017; Valenzuela et al. 2017). The inflammation-associated proteins IL6, CRP, TNF and TGFB1 are also given a central role. Even though TNFRSF11B is not easy to classify, it increases leukocyte adhesion to endothelial cells (Zauli et al. 2007), rendering it an adhesion-related protein. Only FTH is not connected in the STRING-based network. Non-protein biomarkers mentioned alongside the proteins in Table 1 refer to blood cell composition (platelets), glucose and lipids (free fatty acids and lipid hydroperoxides), remnants of coagulation processes (d-dimers, prothrombin fragment) (Friedmann et al. 2019), as well as cardiac markers (natriuretic peptides, copeptin, blood pressure, relating to endothelial/vascular dysfunction) and general stress/health determinants (procalcitonin, cortisol, homocysteine, vitamin D). Most of these non-protein biomarkers are therefore also closely related to inflammation/immunity and to coagulation, and as future work we could envision a network that includes both protein and non-protein biomarkers, which may be based on publication-derived co-mentionings, or correlation data from longitudinal studies.

Our finding that ferritin (FTH1) was not connected in the network of blood-based biomarkers predictive of stroke outcomes deserves comment. In recent studies, ferritin in acute stroke patients was related to baseline rather than long-term disability (Reiche et al. 2019; Alfieri et al. 2020; Garg et al. 2020). It has been shown in the animal model that increased body iron indicated by ferritin worsens ischemic damage induced by transient ischemia and early reperfusion (Garcia-Yebenes et al. 2012). Also, in humans, increased serum ferritin was related to poor outcome after thrombolytic therapy in acute stroke due to early hemorrhagic transformation and severe brain edema (Millan et al. 2007). Extracellular ferritin iron exacerbates the neurotoxic effect induced by glutamate excitotoxicity which plays a crucial role in acute brain ischemia (Millan et al. 2008; Gamez et al. 2021). Together with our STRING network analysis finding, we propose that ferritin mainly influences acute stroke severity, which is already reflected by clinical scores such as the NIHSS, and therefore does not add much value in the prediction of long-term outcome.

## Blood Biomarkers of Stroke Overlap with Biomarkers of Cellular Senescence, Thromboembolism and Sarcopenia, with Links to COVID-19 and Cancer

Given the classification of the biomarkers we presented, it is of interest whether alternative classifications, relating to other biological processes, may be possible. On one hand, the GO annotations in Table 2 further refer to lipid storage (supported only by immuno-inflammatory proteins, though), protein secretion/endocytosis, wound healing, and some other general metabolic processes. On the other hand, we may consider more general biological processes such as sarcopenia, and overlapping biological processes such as cellular senescence (Mankhong et al. 2020). The latter is not frequently mentioned in the literature on predicting ischemic stroke outcome, though there are exceptions (Childs et al. 2017; Valenzuela et al. 2017; Torres-Querol et al. 2021). Our analysis corroborates the evidence for its role in the natural history of stroke-related health deterioration, given that many members of the senescence-associated secretory phenotype (SASP; here: SERPINE1, IL6, CRP, TNF and TGFB1) are placed center stage in the interaction network. At the minimum, we suggest that the inflammatory processes taking place after stroke are expected to be pushed further by senescent cells secreting just the factors that predict health deterioration after stroke. As we suggested elsewhere, cellular senescence may indeed be a driver of the co-morbidity of stroke and (pancreatic) cancer, including thromboembolic events (Henze et al. 2020). Accordingly, a very recent systematic review and meta-analysis (Turner et al. 2021) asked “Is stroke incidence increased in survivors of adult cancers?” and stated that “pancreatic (…); lung (…); and head and neck (…) cancers were associated with significantly increased incidence of stroke.”

**Table 2:**
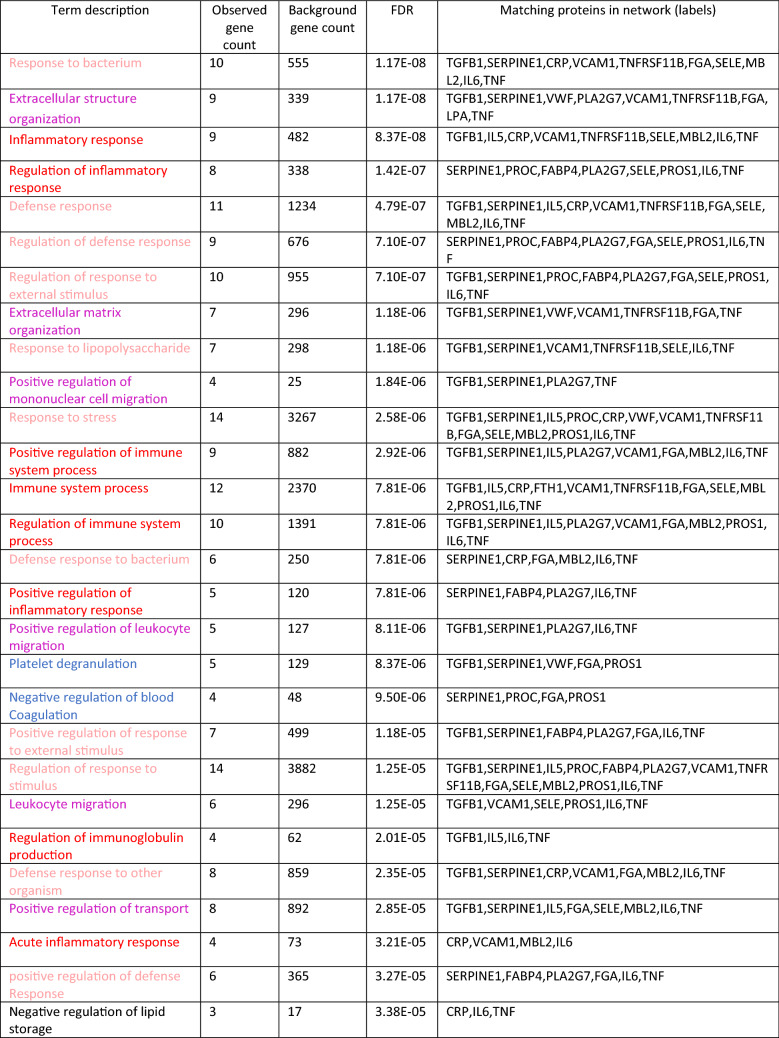

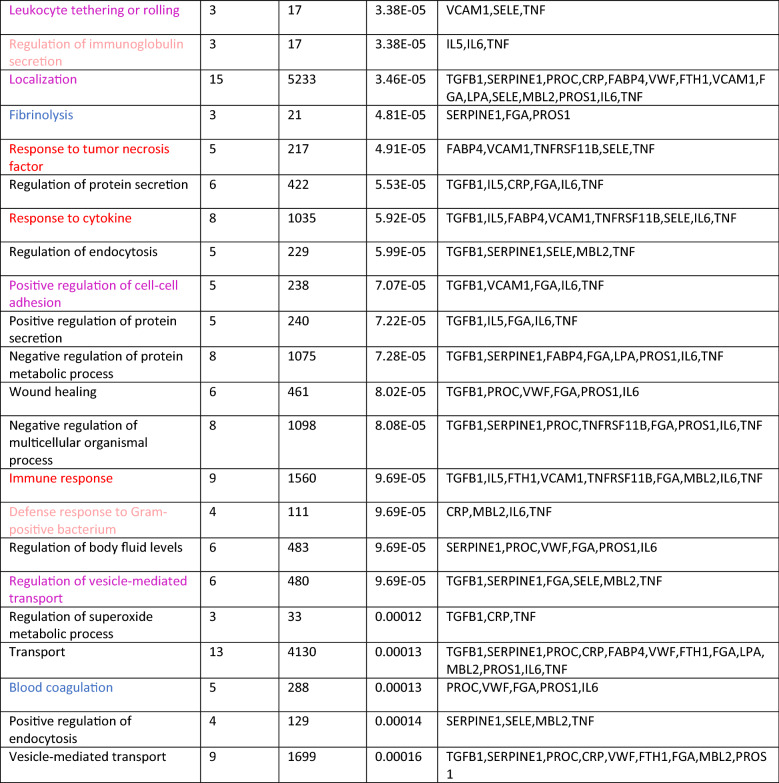
Gene ontology biological process enrichment (first 50 terms) provided by STRING for the network of Fig. 1

The color code of the biomarkers is red (immune-inflammatory), blue (coagulation-related) and magenta (adhesion-related). The pale red color refers to immune-inflammatory processes in a wider sense and black is used for terms that are not assigned. The terms are sorted by FDR (false discovery rate). The first term, response to bacterium, is a known annotation for 10 of the genes from Fig. 1, as listed in the last column, while in the entire universe of all genes, 555 are annotated with that term

In fact, thromboembolism biomarkers in patients with cancer were reviewed recently (Gervaso et al. 2021) and apart from platelet and leukocyte counts, the authors list tissue factor (Coagulation Factor III), d-dimers, soluble P-selectin and CRP, overlapping with the markers of Table 1. While these biomarkers refer to venous thromboembolism, data on biomarkers for arterial thrombotic events specifically in cancer patients are sparse (Gervaso et al. 2021). Very recently, a prospective study described biomarkers in cancer patients with stroke (not necessarily predictive of future outcomes), in stroke patients that do not feature cancer as a comorbidity, and it also contrasts these with another group of patients that are only affected by cancer (Navi et al. 2021); each patient group had size 50. Interestingly, most association biomarkers are valid for stroke patients no matter whether they are also affected by cancer; these are d-dimer, ICAM-1, VCAM-1, thrombomodulin. Only one biomarker (P-selectin) is found associated exclusively in the stroke-only group, and thrombin-antithrombin is found associated exclusively in the cancer-only group. Here, biomarkers were measured around 96 h after the event/diagnosis; multivariable linear regression was used to reveal comparative associations to an outcome defined by prespecified hematological biomarkers and transcranial Doppler microemboli detection, adjusted for race, number of stroke risk factors, smoking, antithrombotics use, and NIHSS. Of interest, all biomarkers of (Navi et al. 2021) are related to coagulation or cell adhesion, and we also note the overlap with the biomarkers of Table 1.

Further, the discrimination between 100 sarcopenia patients and 100 controls was recently demonstrated (Marzetti et al. 2019), based on the proteins MPO, P-selectin, IL8, MCP-1, CRP, MIP-1α, PDGF-BB, and IP-10. Again, there is an overlap with the biomarkers presented in this review, supporting the hypothesis that thrombotic events, including recurrent stroke, as well as sarcopenia may be triggered by post-stroke coagulation and inflammation. A recent transcriptomics analysis highlights the role of the aging immune system in acute stroke (Sykes et al. 2021) and suggests that not just the inflammatory aspects of the immune system play a role in determining disease outcome. However, the functional status of the (adaptive) immune system is not as easy to measure as its contribution to inflammatory processes, explaining why no such markers are prominently reported as protein biomarkers in the blood. Moreover, immunity and inflammation are closely connected in general, and even more so in stroke, considering the immune-cell infiltration usually resulting from the event.

Finally, only recently an overlapping set of proteins (that is, in particular, IL-1α, IL-6, CCL2/MCP-1, MMP9, ICAM-1, PAI-1/SERPINE1, TIMP-1 and TNF-α) and processes (that is, inflammation, coagulation, complement activation, adhesion and senescence) were implicated in COVID-19 pathogenesis, see (Lee et al. 2021) and Box 1. Such an overlap of key factors and processes may suggest that perhaps there is a senescence-based overlap in the pathogenesis of stroke and COVID-19, see also (Dmytriw et al. 2021; Shahjouei et al. 2021).

As the major limitation of any study of this kind, we are limited to the small sets of biomarkers measured a-priori in any of the studies performed thus far, resulting in inspector bias effects. Such a bias may be alleviated by high-throughput omics studies considering transcripts, proteins, lipids or other metabolites. However, transcripts are known to be quite noisy, large-scale proteomics are lacking, and metabolites are not as straightforward in their assignment to biological processes, even though in this review, we consider some of these alongside the proteins. Nevertheless, the most recent transcriptomic analysis (Sykes et al. 2021) highlighted immune-related processes that are closely associated with the observations presented in this review, including interleukin signaling, though on the gene level, they found no transcripts that directly correspond to any of the protein biomarkers in Table 1.

## Discussion

### Key Molecular Blood Markers for Stroke Outcome

Of the blood markers identified here as being relevant for the long-term outcome after stroke, arguably the most important molecular key players are tumor necrosis factor alpha (TNF), interleukin 6 (IL6), fibrinogen (FGA), and plasminogen activator inhibitor-1 (SERPINE1) (Donkel et al. 2019; Lai et al. 2019; Reiche et al. 2019; Alfieri et al. 2020; Lehmann et al. 2021). In the adult brain, TNF is mainly derived from glia, astrocytes, and microglia, and is involved in the regulation of neurotransmitter processes, cell division, blood–brain barrier permeability, leukocyte migration to the central nervous system and angiogenesis (Xue et al. 2022). While increased intra-lesional TNF appears to be beneficial in the acute stroke stage (von Linstow et al. 2021), permanently elevated TNF serum levels may indicate ongoing neuroinflammation driving continued cell death following stroke (Stuckey et al. 2021). IL6 is also involved in the regulation of systemic and local (cerebral intra- and perilesional) inflammatory responses and has recently been identified as an independent predictor of recurrent stroke at 12 months even in patients with persistent adherence to guideline-based secondary stroke prevention (Pan et al. 2021). FGA has a wide spectrum of functions that involve the balance between hemostasis and thrombosis, coagulation and fibrosis, protection from infection and extensive inflammation (Davalos and Akassoglou 2012). In a recent large registry study, high baseline and 90-day FGA levels were associated with increased risk of poor functional outcome and dependence at 1 year after stroke (Hou et al. 2021). SERPINE1 is an endothelial cell-derived paracrine factor belonging to the serine protease inhibitor (serpin) superfamily, a rich source of which is human adipose tissue, contributing directly to an increasing plasma concentration in the obese (Chen et al. 2017). SERPINE1 is increased also in aging and hypertension and has been linked to worse stroke outcomes as a result of poor collateral perfusion, hemorrhagic transformation, and oxidative stress, which could be prevented by the SERPINE1 inhibitor TM5441 in an animal model (Chan et al. 2018). Elevated circulating SERPINE1 was related to increased risk of stroke in patients with atrial fibrillation (Wu et al. 2015). In conclusion SERPINE1 is likely to influence the long-term prognosis after stroke by (i) its acute effects on stroke severity and (ii) its impact on the risk of stroke recurrence.

### Stroke Outcome Biomarkers are Involved in a Variety of Aging-Related Biological Processes

Overall, we suggest that our summary of the outcome biomarkers for ischemic stroke provides evidence for the role of immune‑inflammatory, coagulation-related, and adhesion-related processes in health deterioration and mortality after stroke. The overlap of the outcome biomarkers with the SASP and with thromboembolism biomarkers, as well as the role of senescent cells in blood clotting (Tanaka et al. 2018), suggests that cellular senescence plays a role as well. Cellular senescence is also implicated in sarcopenia (Mankhong et al. 2020), and we thus found an overlap of outcome biomarkers for stroke with sarcopenia biomarkers.

## Conclusions

Aiming for an up-to-date protein biomarker signature that allows a maximum of mechanistic understanding, to predict health deterioration following ischemic stroke, we provided an update of the recent literature, and placed the protein biomarkers into the context of a functional interaction network. The network reconfirmed the long-held proposition that stroke outcome is dependent on the molecular processes of inflammation, coagulation, adhesion and senescence. We also demonstrated some relevant and interesting overlap of these processes, including cellular senescence in particular, with processes associated with cancer, and more specifically with thromboembolism as one of the sequelae of cancer.

For clinical practice, not only predictive accuracy, but also straightforward interpretability as well as measurement cost and convenience can be important considerations for a biomarker signature. To foster interpretability, referring to easily understandable underlying biological processes is of high value. To reduce costs, a focus on a few well-defined biomarkers should be helpful. In the Discussion, we thus suggest a signature of four key biomarkers [TNF alpha, IL6, fibrinogen (FGA) and PAI-1 (SERPINE1)], as a compromise of relevance and utility, which we offer as a starting point for further refinement.

Box 1: Blood-Based Proteins at the Intersection of Inflammation, Coagulation, Complement Activation, Adhesion and Senescence in COVID-19 Severe COVID-19 reflects a system-wide inflammatory response to SARS-CoV-2 infection with significant tissue damage and organ dysfunction in the respiratory tract. We recently marked virus-induced senescence (VIS) as a pivotal stress program evoked by SARS-CoV-2 virus entry into upper airway epithelial cells that triggers an escalating inflammatory downstream cascade (Lee et al. 2021). Senescent respiratory epithelial cells launched a senescence-associated secretory phenotype (SASP) as known from other types of senescence, especially oncogene- or therapy-induced senescence (OIS and TIS, respectively). Of note, equally virus-infected but genetically senescence-incapable cells failed to mount a SASP. Attraction of macrophages to the senescent mucosa induced their secondary, paracrine senescence accompanied with activating M1 polarization. SASP-amplifying macrophages, in turn, contributed as mobile elements to further damage in the lung. Massive elevation of SASP factors became detectable in the serum, characterized by pro-inflammatory cytokines and chemokines, extracellular matrix-active proteases, as well as pro-coagulatory and complement-activating mediators, among them, for instance, IL-1α, IL-6, CCL2/MCP-1, MMP9, ICAM-1, PAI-1/SERPINE1, TIMP-1 and TNF-α (Lee et al. 2021). Key factors detected in this study appear to largely overlap with biomarkers listed in Table 1 in the context of ischemic stroke, notably IL-6, PAI-1/SERPINE1 and TNF-α, suggesting an overlap of the molecular processes related to ischemic stroke on one hand and to COVID-19 on the other hand. Importantly, early intervention upon SARS-CoV-2 infection with a variety of senolytic agents, i.e., drugs that selectively eliminate senescent cells, mitigated histopathologic features of lung affection to varying extents and dramatically reduced blood-based protein representatives of systemic inflammation, thereby confirming VIS as a critical driver and therapeutic target in COVID-19.

## Data Availability

Data sharing is not applicable to this article as no datasets were generated or analyzed during the current study.

## Abbreviations

GO: The gene ontology (GO) is a major bioinformatics initiative to unify the representation of gene and gene product attributes across all species
HUGO: Human Genome Organization
Modified Rankin Scale: The modified Rankin Scale (mRS) is a commonly used scale for measuring the degree of disability or dependence in the daily activities of people who have suffered a stroke or other causes of neurological disability
NIHSS: The National Institutes of Health Stroke Scale (NIHSS) is a tool used by healthcare providers to objectively quantify the impairment caused by a stroke
NLR: Neutrophil-lymphocyte ratio
STRING: A database of known and predicted protein-protein interactions. The interactions include direct (physical) and indirect (functional) associations; they stem from computational prediction, from knowledge transfer between organisms, and from interactions aggregated from other (primary) databases
WBC: White-blood cell count
